# Antifungal Activity of Natural Volatile Organic Compounds against Litchi Downy Blight Pathogen *Peronophythora litchii*

**DOI:** 10.3390/molecules23020358

**Published:** 2018-02-08

**Authors:** Mengyu Xing, Li Zheng, Yizhen Deng, Dandan Xu, Pinggen Xi, Minhui Li, Guanghui Kong, Zide Jiang

**Affiliations:** 1 Department of Plant Pathology/Guangdong Province Key Laboratory of Microbial Signals and Disease Control, South China Agricultural University, Guangzhou 510642, China; xingmengyu@hainu.edu.cn (M.X.); bluestar183@163.com (L.Z.); Dengyz@scau.edu.cn (Y.D.); happyxudandan@126.com (D.X.); xpg@scau.edu.cn (P.X.); liminhui@scau.edu.cn (M.L.); gkong@scau.edu.cn (G.K.); 2 Institute of Tropical Agriculture and Forestry, Hainan University, Haikou 570228, China; 3 Chinese Academy of Tropical Agricultural Sciences Guangzhou Experimental Station, Guangzhou 510140, China

**Keywords:** *Streptomyces fimicarius* BWL-H1, volatiles, *Peronophythora litchii*, litchi downy blight

## Abstract

Litchi (*Litchi chinensis* Sonn.) is a commercially important fruit but its production and quality are restricted by litchi downy blight, caused by the oomycete pathogen *Peronophythora litchii* Chen. Volatile substances produced by a biocontrol antinomycetes *Streptomyces fimicarius* BWL-H1 could inhibited *P. litchii* growth and development both in vitro and in detached litchi leaf and fruit infection assay. Transmission Electron Microscopy (TEM) and Scanning Electron Microscopy (SEM) analyses indicated that volatile organic compounds (VOCs) from BWL-H1 resulted in severe damage to the endomembrane system and cell wall of *P. litchii* cells in vitro and abnormal morphology of appressoria, as well as deformed new hyphae in infection process. VOCs could suppress mycelial growth, sporulation, while with no obvious effect on sporangia germination. Based on gas chromatography-mass spectrophotometric analyses, 32 VOCs were identified from *S. fimicarius* BWL-H1, the most abundant of which was phenylethyl alcohol. Eight VOCs, including phenylethyl alcohol, ethyl phenylacetate, methyl anthranilate, α-copaene, caryophyllene, humulene, methyl salicylate and 4-ethylphenol, that are commercially available, were purchased and their bioactivity was tested individually. Except for humulene, the other seven tested volatile compounds shown strong inhibitory activity against mycelial growth, sporulation, sporangia germination and germ-tube growth of *P. litchii*. Especially, 4-ethylphenol showed the highest inhibitory effect on sporulation at a very low concentration of 2 µL/L. Overall, our results provided a better understanding of the mode of action of volatiles from BWL-H1 on *P. litchii*, and showed that volatiles from BWL-H1 have the potential for control of postharvest litchi downy blight.

## 1. Introduction

Litchi (*Litchi chinensis* Sonn.) is one of the most popular and important subtropical fruits due to its taste, appearance, and economic value [1]. Litchi fruit pulp is rich in essential amino acids, vitamins, trace elements and flavonoids that are beneficial for humans, and therefore it is consumed as a fresh fruit, or is used in China in the production of traditional Chinese medicines. However, litchi quality and yield are greatly limited by the plant diseases. Litchi downy blight, caused by *Peronophythora litchii* Chen, is the most severe disease in litchi. *P. litchii* can infect litchi plants at a broad range of growth stages and tissues including tender leaf, flower, and mature fruit period, and even extends to storage and transport at the postharvest stage. *P. litchii* infects litchi leaves or fruits through oospores, which subsequently develop into sporangia and release zoospores in the litchi orchard. The oomycete infection initially causes withering and watery brown spots on the infection sites of tender leaf or fruit, or panicle rot, and finally produces downy white sporangiophores during late infection, which leads to major pre- and postharvest decay of the fruit and results in considerable economic losses [2,3]. A wide range of chemical fungicides have been tested and evaluated to control this disease [4], but considering of the unavoidable emergence of fungicide resistance as well as toxicological risks and environmental pollution [5], alternative means to control litchi downy blight are urgently required.

Microbial biocontrol agents, as important alternatives to fungicides to control postharvest diseases, have attracted much more attention recently. Increasing interest is being devoted to the use of volatile organic compounds produced by microorganisms, such as the endophytic fungus *Muscodor albus* [6,7,8] and yeast *Candida intermedia* [9], antagonistic bacteria *Bacillus* strains [10,11] and *Streptomyces* spp. [12,13,14]. Antifungal volatile organic compounds (VOCs) can be used to control plant pathogens because of its low molecular weight and polarities, and easily diffuse through the porous structure of soil and over great distances in the atmosphere. Previous studies have reported that the volatiles from actinomycetes caused severe morphological alterations on the conidiophores and hyphae of several fungi [15], and inhibit conidial germination of *Cladosporium cladosporioides* [16]. Wan et al. [12] demonstrated that volatiles produced by *Streptomyces* showed antifungal activity on several plant diseases, such as seedling blight of rice, leaf blight of oilseed rape and fruit rot of strawberry. VOCs of *Streptomyces globisporus* JK-1 exerted antifungal activity against *Botrytis cinerea* on tomato fruit [17] and the VOCs of *Streptomyces coelicolor* showed strong inhibitory activity on the mycelial growth and spore germination of *B. cinerea* and *Penicillium chrysogenum* [18]. Moreover, Wang et al. [19] reported that VOCs from *Streptomyces philanthi* damaged the mycelial membrane permeability of *Fusarium moniliformei.* However, no information about the effect of VOCs produced by the biocontrol strains on the growth and pathogenicity of *P. litchii* could be found.

In this study we assessed antifungal activity of volatiles from *S. fimicarius* BWL-H1 on *P. litchii* in vitro and in vivo, and identified several effective components from these volatile substances. The action mechanism was investigated by Electron Microscopy (EM) analyses. The results displayed that volatiles from *S. fimicarius* BWL-H1 have the potential for control of postharvest litchi downy blight.

## 2. Results

### 2.1. Antifungal Activity of S. fimicarius BWL-H1 Volatiles on P. litchii

**Figure 1:**
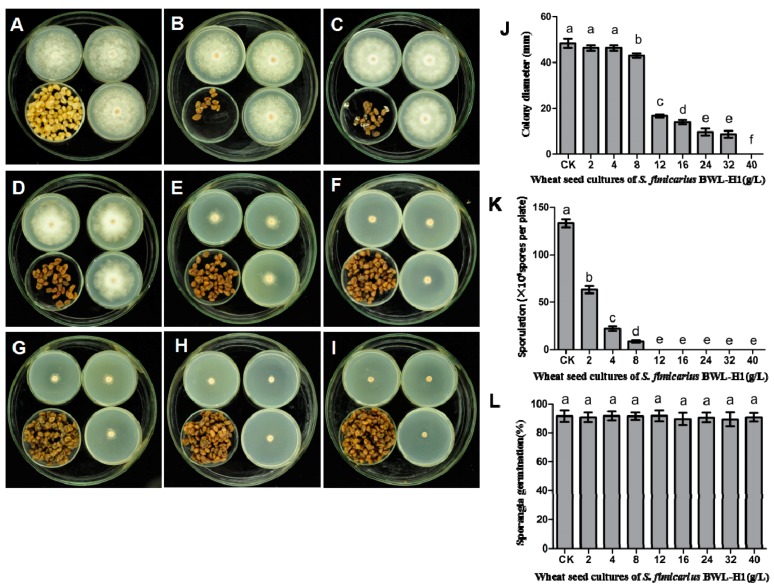
Suppression of *P. litchii* mycelial growth, sporangial production and germination by *S. fimicarius* BWL-H1volatiles. *P. litchii* was fumigated with 16 g/L of wheat seeds without *S. fimicarius* BWL-H1 culture (**A**); or with various amounts (2, 4, 8, 12, 16, 24, 32, 40 g/L) of wheat seed culture of *S. fimicarius* BWL-H1 (**B**–**I**) for 6 d and photographed. Barchart represents mean ± S.E. of colony diameter (**J**); sporangial production (**K**); or percentage of sporangia germination (**L**); grown as in (**A**–**I**). CK represents unfumigated control as shown in (**A**). For each instance, three independent experiments with triplicates were performed and values followed by different letters were significantly different (*p* < 0.05).

The effect of volatiles produced by wheat seed culture of *S. fimicarius* BWL-H1 was tested in a sealed Petri dish chamber, and the result showed that radial mycelial growth of *P. litchii* was greatly suppressed by the volatiles in a dose-dependent manner. After 6 d in the presence of (2, 4, 8, 12, 16, 24, 32 g/L) wheat seed culture of BWL-H1, the mycelial growth (include the 5 mm diameter of the inoculum plug) was 46.3, 46.1, 43.2, 16.7, 14.2, 9.7, 8.7 mm, respectively. No mycelial growth was observed in the presence of volatiles produced by 40 g/L BWL-H1 cultures (Figure 1A–J), whereas autoclaved wheat seed displayed no observable effects on radial mycelial growth. The sporulation of *P. litchii* was also suppressed by the treatment of BWL-H1 cultures with a dose-dependent manner, and no sporangia were observed after treated with BWL-H1 culture more than 12 g/L (Figure 1K), However, mycelial growth was not completely suppressed at 12–32 g/L of BWL-H1 culture. In addition, sporangial germination was not affected by such fumigation treatment (Figure 1L).

**Figure 2:**
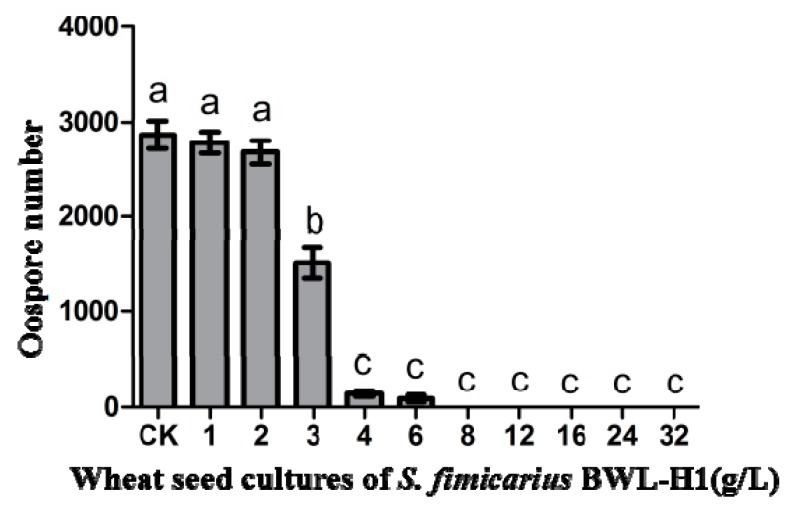
Suppression of *P. litchii* oospore production by *S. fimicarius* BWL-H1 volatiles. Barchart represents mean ± S.E. of oospores per cm^2^ area from 10 mm around the inoculation site. Data represent three independent experiments with triplicates. Values followed by different letters were significantly different (*p* < 0.05).

Then, the effect of VOCs on oospore production was examined. After 14 d of incubation at 25 °C, the production of oospore significantly reduced when treated with BWL-H1 cultures and no oospore was found from 8 g/L to 32 g/L of wheat seed cultures (Figure 2).

Overall, our results displayed *S. fimicarius* BWL-H1 VOCs suppressed mycelial growth, sporulation and oospore production of *P. litchii*.

### 2.2. VOCs Treatment Caused Morphological and Ultrastructure Alteration on P. litchii

**Figure 3:**
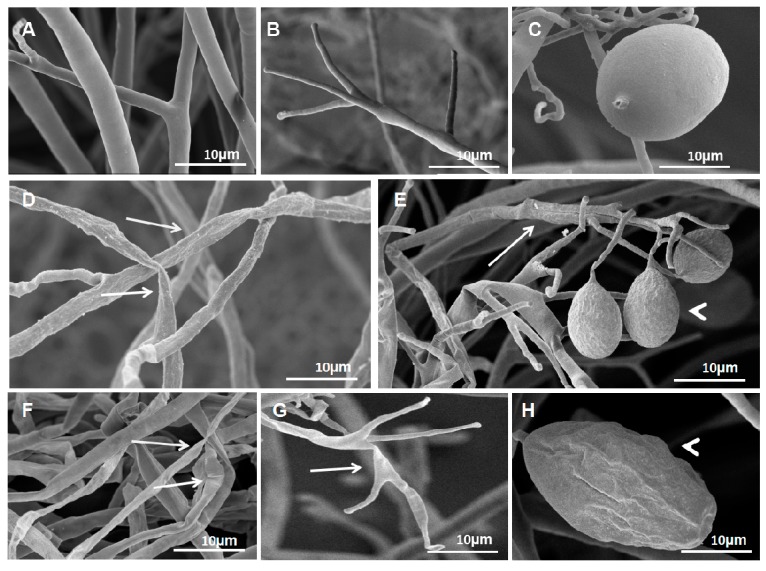
*S. fimicarius* BWL-H1 volatiles caused morphological changes in *P. litchii* hyphae, sporangiophores and sporangia. (**A**–**C**) untreated control; (**D**–**E**) culture fumigated with 40 g/L wheat seed culture of *S. fimicarius* BWL-H1; (**F**–**H**) culture fumigated with 100 g/L wheat seed culture of *S. fimicarius* BWL-H1. Arrows denote shrinking or distorted mycelia and sporangiophores, and arrowhead for deformed sporangia.

To understand the mechanism of action of VOCs on *P. litchii*, the morphology of *P. litchii* mycelia and sporangiophores treated with VOCs was examined by Scanning Electron Microscopy (SEM). The untreated *P. litchii* culture showed straight and uniform mycelia and sporangiophores (Figure 3A,B), as well as oval, plump and homogenous sporangia (Figure 3C). However, the fumigated *P. litchii* culture (40 g/L BWL-H1 culture) showed collapsed and shrunken mycelia and sporangiophore (Figure 3D,E, arrows), and a warty surface of sporangia (Figure 3E, arrowhead). Higher concentration of fumigation (100 g/L BWL-H1 culture) led to much more severe morphological changes on the mycelia and sporangiophore, with the appearance of swelling and malformation mycelia, sporangiophore (Figure 3F,G, arrow) and severely collapsed sporangia (Figure 3H, arrowhead).

**Figure 4:**
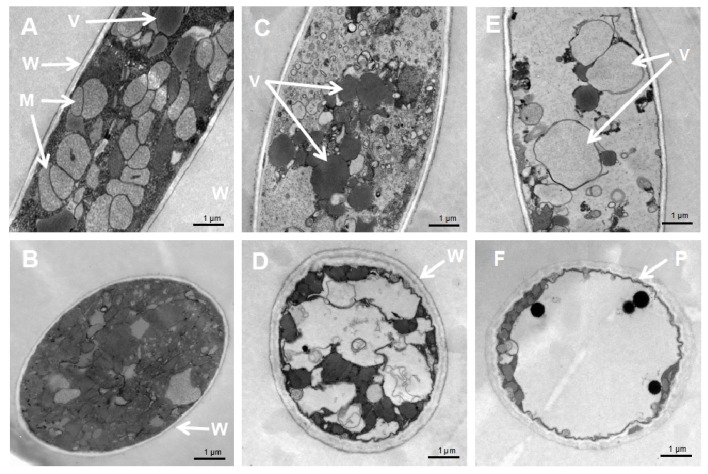
Cellular damages caused by *S. fimicarius* BWL-H1 volatiles. (**A**,**B**) untreated control; (**C**,**D**) mycelial samples fumigated with 40 g/L wheat seed culture of *S. fimicarius* BWL-H1; (**E**,**F**) mycelial samples fumigated with 100 g/L wheat seed culture of *S. fimicarius* BWL-H1. (**A**), (**C**) and (**E**) were longitudinal section through *P. litchii* hyphae, while (**B**,**D**,**F**) were tangential section. M, mitochondria; P, plasma membrane; V, vacuoles; and W, cell wall.

Transmission Electron Microscopy (TEM) observation showed that the untreated hyphae of *P. litchii* contained uniform cytoplasm and several vacuoles (Figure 4A). The cell wall and the plasma membrane were intact and with a uniform shape, and the mitochondria also appeared with normal morphology (Figure 4A,B). The VOC treatment (40 g/L BWL-H1) led to thickened cell walls and plasma membrane, as well as disruption in endomembrane systems including mitochondria membranes, vacuoles and organelles that were injured or even were destroyed (Figure 4C,D). Higher amounts of wheat seed culture of BWL-H1 caused even greater destruction of intracellular components, vacuolation was increased and damaged plasma membranes were prominent (Figure 4E,F).

### 2.3. S. fimicarius BWL-H1 Volatiles Repressed P. litchii Infection on Litchi Leaves

**Figure 5:**
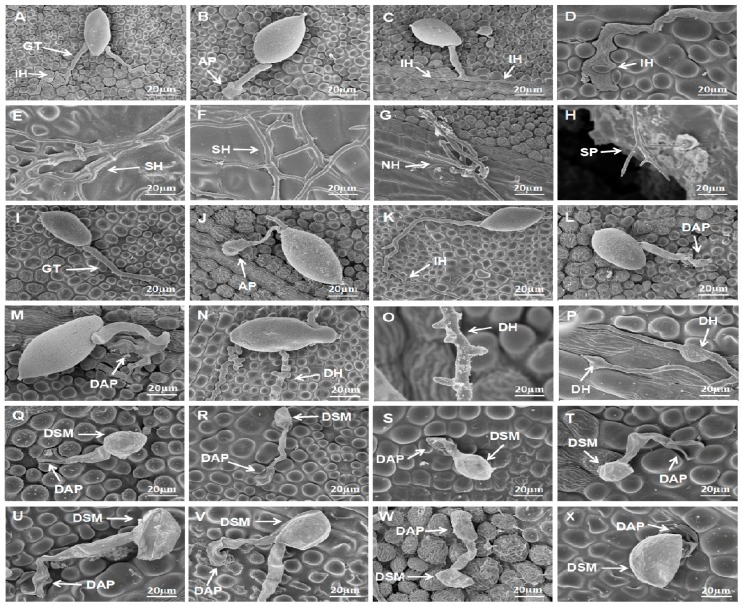
Effect of *S. fimicarius* BWL-H1 volatiles in *P. litchii* infection process. Inoculation of *P. litchii* on litchi leaves, without volatiles fumigation, were sampled at 3 h (**A**,**B**); 6 h (**C**); 9 h (**D**); 12 h (**E**); 24 h (**F**); and 48 h (**G**,**H**), and imaged. Fumigated with 12 g/L wheat seed culture of *S. fimicarius* BWL-H1, the inoculated leaves were sampled and imaged at 3 h (**I**,**J**); 6 h (**K**); 9 h (**L**); 12 h (**M**); 24 h (**N**); and 48 h (**O**,**P**). Inoculation of *P. litchii* on litchi leaves fumigating with 16 g/L wheat seed culture of *S. fimicarius* BWL-H1 were imaged at 3 h (**Q**,**R**); 6 h (**S**); 9 h (**T**); 12 h (**U**); 24 h (**V**); and 48 h (**W**,**X**). SEM images for each instance were representative of three independent biological replications. GT, germ tube; IH, infection hyphae; SH, secondary hyphae; NH, new hyphae; AP, appressorium; DAP, deformed appressorium; SP, sporangiophore; DSM, deformed sporangia; DH: deformed hyphae.

SEM observation indicated VOCs effectively suppressed the infection of *P. litchii* on litchi leaves. In the control treatment, *P. litchii* sporangia germinated to form germ tubes or appressoria and infected the host at 3 h post inoculation (hpi), and formed infection hyphae at 6–9 hpi (Figure 5A–D). Long secondary hyphae were visible in the inoculation site at 12 hpi and 24 hpi (Figure 5E,F). Abundant newly formed hyphae emerged far from inoculation site, and at 48 hpi, sporangiophores and sporangia produced from the new hyphae (Figure 5G,H).

In the treatment of leaves fumigated with 12 g/L wheat seed culture of *S. fimicarius* BWL-H1, *P. litchii* sporangia were able to germinate at 3 hpi (Figure 5I,J), but rare infection hyphae were visible at 6 hpi (Figure 5K). In addition, appressoria with abnormal morphology were visible at 9–12 hpi (Figure 5L,M) and deformed secondary hyphae were visible at 24–48 hpi (Figure 5N–P). When the inoculated leaves were fumigated with 16 g/L wheat seed culture of *S. fimicarius* BWL-H1, sporangia and appressoria were badly destructed, abundant sporangia and appressoria were collapsed and shrunken, and most of the deformed appressoria unable to penetrate the litchi leaves epidermis (Figure 5Q–X). Quantification of invasive hyphae (IH) for both *P. litchii* inoculation on un-treated or treated leaves, and for deformed structures for *P. litchii* inoculation on treated leaves, were presented as in Table 1. Such deformed structures were rarely seen in the inoculation on un-treated leaves and therefore was not quantified.

Overall, results of detailed time-coursed SEM analysis showed that *S. fimicarius* BWL-H1 VOCs effectively repressed *P. litchii* infection by causing damage to appressria and subsequently suppressing penetration and invasive hyphae formation.

### 2.4. S. fimicarius BWL-H1 Volatiles Prevented Litchi Downy Blight In Vivo

**Figure 6:**
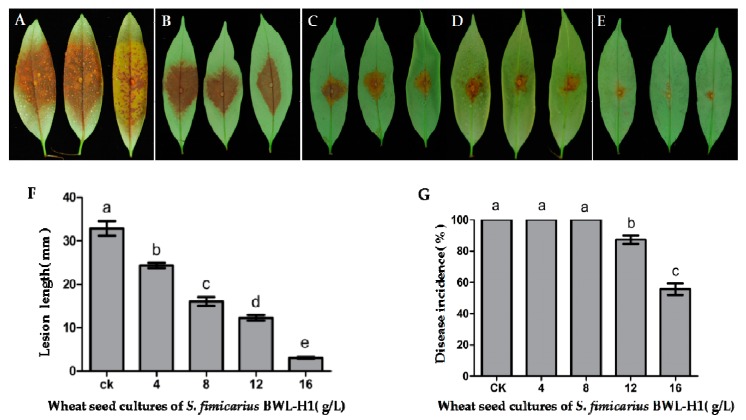
Antifungal activity of VOCs against *P. litchii* on detached leaves. (**A**) unfumigated control; (**B**–**E**) *P. litchii* inoculated leaves were fumigated with various amounts (4, 8, 12, 16 g/L) of wheat seed culture of *S. fimicarius* BWL-H1 volatiles; (**F**,**G**) Lesion length and Disease incidence were measured at 48 h post inoculation. Mean ± S.E. was based on 3 replicates. Values followed by different letters were significantly different (*p* < 0.05).

Litchi downy blight is prevalent throughout the growth and development of litchi trees, especially during the tender leaf, flower and mature periods, causing exfoliation of leaves and flower, and fruit decay, leading to significant economic losses. Hence, we tested the effectiveness of VOCs on the control of litchi downy blight using litchi leaves and fruits. The efficacy of VOCs on preventing litchi downy blight on litchi leaves and litchi fruits were conducted by fumigating with VOCs. Inoculation results on litchi leaves showed that disease severity was significantly decreased followed with the increasing amount of BWL-H1 culture. Lesion diameter and Disease incidence significantly decreased after treated with the BWL-H1 VOCs, when the leaves were fumigated by the wheat seed culture of BWL-H1 (ranging from 12 to 16 g/L), the mean lesion length was decreased from 12.3 to 3.1 mm, and the disease incidence was decreased from 87.3% to 55.7%. However, unfumigated leaves were severely infected, the disease incidence reached 100% and the mean lesion length reached 32.9 mm (Figure 6A–G).

**Figure 7:**
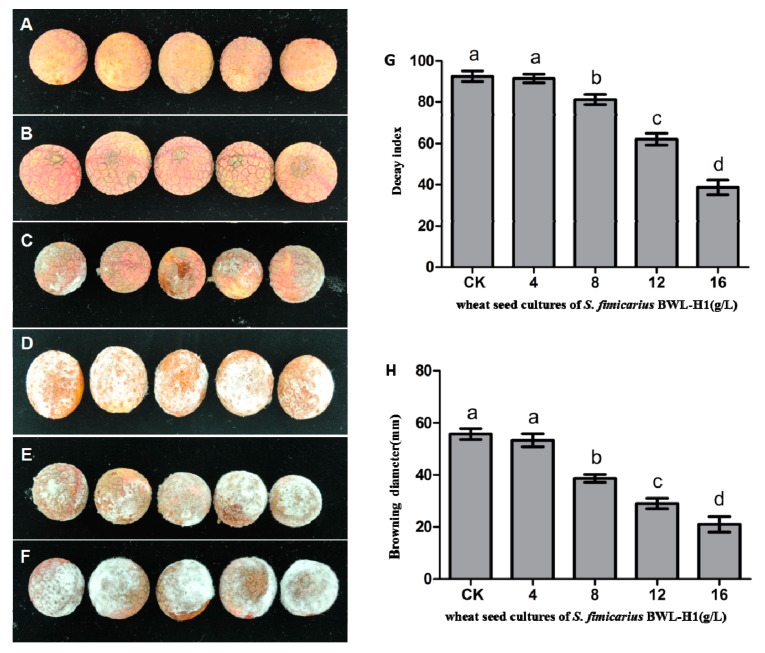
Antifungal activity of VOCs against *P. litchii* on fruits. (**A**) Negative control (without *P. litchii* inoculation); (**B**–**E**) *P. litchii* inoculated fruits fumigated with 16, 12, 8, 4 g/L of wheat seed culture of *S. fimicarius* BWL-H1 volatiles; (**F**) unfumigated control, with autoclaved wheat seeds. Each treatment consisted of three replicates with 30 fruits in each replicate; (**G**,**H**) Decay index and browning diameter of harvested litchi fruits inoculated with *P. litchii* after 5 d storage at 25 °C. Data are the mean ± S.E. from three replicates per treatment.

For disease prevention on litchi fruits, the results showed that fumigating with BWL-H1 VOCs substantially reduced the disease severity, especially at higher VOCs concentrations (Figure 7B–F) compared with the non-inoculated controls (Figure 7A). After 5 d of storage at 25 °C, the decay indices of fruit fumigated with 8–16 g/L wheat seed culture of BWL-H1 were significantly lower than that of unfumigated infected fruits (Figure 7G). Peel browning of postharvest litchi fruits caused by litchi downy blight greatly decreased after VOCs fumigation (Figure 7A–C). After incubation for 5 d. at 25 °C, the browning diameter of the fruits fumigating with 8–16 g/L wheat seed culture of BWL-H1 were significantly smaller than that of unfumigated control (Figure 7H). VOCs fumigation showed no obvious effect on the color and taste of litchi fruit pulp. The results suggested that BWL-H1 VOCs could effectively prevent litchi downy blight.

### 2.5. Identification and Verification of Effective VOCs Produced by S. fimicarius BWL-H1

By GC-MS analysis, there were 12 and 21 volatile organic compounds were identified from 14 d and 21 d wheat seed culture of *S. fimicarius* BWL-H1, which all had high similarity indices (SI) > 90% with ones from the NIST library. These compounds mainly included alkenes, esters, alcohols, and phenols (Table 2 and Table 3). Most of the volatiles from the 14 d and 21 d cultures of *S. fimicarius* BWL-H1 were generally similar but differed in quantity. Among those, phenylethyl alcohol, ethyl phenylacetate, caryophyllene, methyl salicylate, 4-ethylphenol and humulene, were found in the volatiles from 21 d cultures, but not in the volatiles from 14 d cultures, and their quantities is larger than other compounds from the volatiles of 21 d cultures. In addition, methyl anthranilate, and α-copaene were detected in larger quantities (up to 10-fold-greater) in 21 d than in 14 d cultures (Table 2 and Table 3).

In preliminary inhibition tests against fungi, the volatiles from 21 d cultures showed stronger antifungal activity than 14 d cultures (Figure S1). Therefore, these particular volatile compounds were selected for antifungal activity test. Among the eight tested volatile compounds (Table 4), humulene displayed no significant effect on mycelial growth or sporulation, but with weak inhibitory activity on sporangia germination and the growth of germ tube at the concentration of 200–1000 µL/L (Table 5, Table 6, Table 7 and Table 8). The other seven volatile compounds showed strong inhibitory activity against mycelial growth, sporulation, sporangia germination and the growth of germ tube, especially, 4-ethylphenol almost completely inhibited sporangium formation from concentration as low as 2 µL/L onwards (Table 5, Table 6, Table 7 and Table 8).

## 3. Discussion

Microbes represent an alternative and promising strategy for plant disease control in agricultural practice as biocontrol agents against fungal or oomycete plant pathogens. It has been reported that VOCs from several species of *Streptomyces* genus, including *S. globisporus* JK-1 [13,17], *S. coelicolor* [18], *S. philanthi* RM-1-138 [20], *S. alboflavus* TD-1 [19], possess fungicidal activity, causing defects in mycelial growth, sporulation and spore germination of various fungal plant pathogens in vitro. In this study, we verified the antifungal activity of VOCs from *S. fimicarius* BWL-H1, against *P. litchii* and the results indicated VOCs significantly suppressed *P. litchii* in vitro, which consistent with the fungicidal activity of *S. globisporus* JK-1 VOCs against *Penicillium italicum* [13]. However, *S. fimicarius* BWL-H1 VOCs displayed no obvious effects on sporangia germination, which was different from the previous study that *S.* spp. volatiles showed inhibit activity on spore germination of *P. italicum*, *C. cladosporioides*, and *F. moniliforme* [13,16,19,21]. The possible reason could be that the sporangia of *P. litchii* germinate within 2 h, during which time the concentration of VOCs from BWL-H1 becomes low and not enough to inhibit sporangial germination. To our knowledge, this is the first report on the antifungal properties of VOCs from a biocontrol bacterium against oomycete plant pathogen *P. litchii*.

We performed detailed electron microscopy of *P. litchii* fumigated with VOCs provided clues to the action mechanism of BWL-H1 VOCs on inhibitting *P. litchii* growth and development. SEM analysis showed that BWL-H1 VOCs caused deformation and concave collapses of *P. litchii* hypha, which was consistent with the SEM observation. TEM analysis displayed that the plasma membrane of the hyphae were severely damaged and certain intracellular organelles disappeared. These results were similar to the damages on *P. litchii* induced by hypothemycin, zeamines or isoliquiritin [22,23,24] or on *B. cinerea* and *Lophodermium seditiosum* induced by methyl *cis*-7-oxo-deisopropyl dehydroabietate [25].

Thirty two volatile compounds from *Streptomyces fimicarius* BWL-H1 were identified by GC-MS analysis. Phenylethyl alcohol was found to be the most abundant component. Previous studies showed that phenylethyl alcohol has been found in an endophytic fungus *Muscodo ralbus*, Streptomyces spp., and *Bacillus amyloliquefaciens* SQR-9 [12,13,26,27,28], Other identified compounds, such as caryophyllene and α-copaene, have been reported to be components of antimicrobial volatiles in the essential oil of basil (*Ocimum basilicam*) and *C. Macrolepis* var. formosana Florin leaf collected from botanical sources [29,30,31]. Methyl salicylate was reported to induce systemic resistance in tobacco plants [32]. However, significant difference was noticed in terms of the composition of individual volatile compounds from *S. fimicarius* BWL-H1 and from some other actinomycetes [13,33,34]. Thus different organisms and different species may release similar or different types of volatile substances, with a variety of effects. In this study, seven individual volatile compounds showed prominent antifungal activity against *P. litchii* in vitro. This is the first report on the antifungal activity of ethyl phenylacetate, methyl anthranilate, α-copaene, methyl salicylate and 4-ethylphenol against a postharvest disease. However, the possibility of synergistic effects of individual compounds in mixture and action mechanism remains largely unknown.

In conclusion, our research indicated that the VOCs produced by a biocontrol strain *S. fimicarius* BWL-H1 exhibited excellent antifungal activity against *P. litchii*. Furthermore, the greater the amount of wheat seed culture of BWL-H1, the greater the inhibitory activity against *P. litchii* both in axenic culture and in planta. Overall, this study provides useful clues on the antifungal mechanism of VOCs from a bio-control bacterium, which may be useful for prevention and control of litchi fruits decay and browning caused by *P. litchii* infection during storage and transportation.

## 4. Materials and Methods

### 4.1. Microorganisms and Cultural Media

BWL-H1 was screened from 476 strains isolated from soil under wild litchi tree of Bawangling Primeval Forest Reserve in Hainan Province, China, and identified as *Streptomyces fimicarius* by characterization of its morphological and physiological features (M.Y. Xing and Z.D. Jiang, unpublished data) and comparison with its 16S rDNA sequence (GenBank Accession No. MG197994) with those available in NCBI website. The oomycete pathogen *P. litchii* was cultured on potato dextrose agar (PDA) at 25 °C [35]. For production of volatile substances, BWL-H1 was cultured in potato sugar broth at 28 °C, shaking at 180 rpm for 5–7 d. Then, a spore suspension of BWL-H1 diluted from broth culture to the concentration of 1 × 10^7^ spores/mL was inoculated on autoclaved wheat seeds in conical flasks (1000 mL), at 1 mL per 100 g of wheat seeds.

### 4.2. Plant Materials

Tender litchi leaves (cv. Huaizhi) were obtained from litchi orchard in the South China Agricultural University (Guangzhou, China). All leaves were selected with uniform shape and size as well as disease-free. All leaves were random divided into five groups and 30 leaves in each group for the experiments. Litchi fruit (cv. Huaizhi) at about 80% maturation were harvested from a commercial orchard in Conghua, Guangzhou, China. All fruits were selected with uniform shape, color and size as well as disease-free. All fruits were random divided into six groups and 30 fruits in each group for the experiments.

### 4.3. Antifungal Activity Assay

An antifungal bioassay followed with the method described by Li et al. [13], as four small Petri dish bottoms (60 mm diameter and 15 mm tall) were placed inside a larger Petri dish (150 mm diameter and 30 mm tall with 0.5 L inner volume), three small dishes contained 5 mL of PDA and were inoculated with *P. litchii*, and the fourth contained different weights of wheat seed culture of BWL-H1 (varied from 2 g/L to 40 g/L) in each treatment container. Non-BWL-H1-inoculated wheat seeds were used as blank control. The large dishes were sealed with Parafilm which allowed for free gas exchange between colonies while preventing direct contact, and incubated at 25 °C for 6 d before measurement of colonial diameter of *P. litchii* cultures. The total number of the sporangia of each plate was also assessed. For each treatment, three independent replicates were performed and the experiment was repeated for three times.

Sporangia suspensions (5 × 10^4^ sporangia/mL) of *P. litchii* were harvested from 5 d old cultures. In three smaller dishes containing 3 mL of PDA, 2 μL droplets of sporangia suspension (5 × 10^4^ sporangia/mL) of *P. litchii* were placed on the PDA in these smaller plates, each plate with five points, and then they were treated with VOCs as described above. After 2 h of incubation at 25 °C, 200 sporangia per replicate were observed under a high-end microscope (DMi8, Leica, Wetzlar, Germany) to count both germinated and nongerminated sporangia. The sporangia germination percentage was calculated. A sporangium was considered germination when the germ tube length was 1.5 times the sporangium diameter.

To determine the suppression on oospores production, *P. litchii* was inoculated on carrot agar (CA) medium in Petri dishes (60 mm diameter), and fumigated by the VOCs produced from different amounts of wheat seed culture of BWL-H1, varying from 1 g to 32 g of culture per liter of airspace in treatment containers. Non-BWL-H1-inoculated wheat seeds were used as blank control. The fumigation plates were inoculated for 14 d in dark. Then, plugs (1 cm × 1 cm) were cut from 10 mm around inoculation site, oospore numbers were counted under microscope. For each treatment, three independent replicates were performed and the experiment was repeated for three times.

To study the effects of selected individual volatile compounds on mycelial growth, similar bioassay described above was used, while the fourth dish contained a piece of autoclaved filter paper (square, 20 mm × 20 mm), to which one of the select compounds was added. 2 μL/L to 1000 μL/L of individual volatile compounds, with equal amount of sterile distilled water as control, were incubated in a sealed petri dish chamber. After 6 d, colony diameter and sporangia number were assessed. The effect of select compounds on sporangia germination and sporangia germ tube length was also assessed using the antifungal bioassay described above. The experiment was repeated for three times.

### 4.4. Scanning Electron Microscopy (SEM) and Transmission Electron Microscopy (TEM)

To examine the effect of VOCs on the ultrastructure of *P. litchii* in vitro, a dual cultures chamber was made as follows: two petri dishes were stacked, one of which was with 40 g/L or 100 g/L wheat seed culture of *S. fimicarius* BWL-H1, and the other with a plug of 3-d cultured *P. litchii* mycelium (at 25 °C on CA medium). The stacked dishes were sealed with Parafilm to obtain a dual cultures chamber with 200 mL of airspace. The sealed double-dish chamber was incubated for 3 d at 25 °C, before electron microscopy.

To examine the antifungal effect of VOCs on infected (detached) litchi leaves, 10 leaves (back-to-front) were placed on the wet filter paper (180 mm diameter) in a Petri dish (200 mm diameter, 30 mm high), with a smaller Petri dish (90 mm-diameter) containing wheat seed culture of BWL-H1 in the center, to form a fumigation chamber. Leaves were inoculated with 2 μL of sporangia suspension (2 × 10^4^ sporangia/mL) in the abaxial side. The amount of wheat seed culture of BWL-H1 ranged from 12 g/L to 16 g/L, and non-BWL-H1-inoculated wheat seeds were used as blank control. The leaf samples (8 mm × 5 mm in size) were collected at various time intervals (3, 6, 9, 12, 24, 48 h after treatment) and subject to SEM analysis.

The effect of volatiles on the ultrastructure of *P. litchii* was examined by SEM and TEM respectively. Sample preparation was according to the method described by Liao et al. [22]. The SEM samples were sputter-coated with gold palladium (JEE420, NTC, Nanto, Japan) and examined with a LEO-1530VP scanning electron microscope (LEO, Oberkochen, Germany) operated at 5 kV. A transmission electron microscope (Tecnai, FEI, Hillsboro, OR, USA) at 100 kV, was used for TEM analysis.

### 4.5. Plant Inoculation Bioassay

The detached litchi leaves were placed in the Petri dish and inoculated with sporangia suspension of *P. litchii*. All infected leaves were treated with different amount of wheat seed culture of BWL-H1 (4–16 g/L) and non-BWL-H1-inoculated wheat seeds were used as blank control. Lesion diameter and infection rate were measured at 48 h post inoculation. Each treatment included three replicates and with 30 leaves in each replicate. The litchi fruits inoculated with 10 μL of sporangia suspension (2 × 10^4^ sporangia/mL) were incubated at 25 °C. Fruit decay and peel browning was assessed. Each treatment included three replicates and with 30 fruits in each replicate.

### 4.6. Analysis of VOCs produced by BWL-H1

Analysis of VOCs was performed using established procedues [36,37], with minor modification. Volatiles from 14 d and 21 d wheat seed cultures of BWL-H1 were collected using the solid-phase microextraction (SPME) fiber (Supelco, Palo Alto, CA, USA) metal alloy (PDMS 100 μm). 4 g of autoclaved wheat seeds (as blank control) or wheat seed culture of BWL-H1 in a 20 mL vial was sealed with aluminum foil and incubated at 25 °C for 12 h, before inserting the SPME fiber into the vial and incubated at 25 °C for another 30 min. The volatile compounds absorbed on the fiber were desorbed at 250 °C for 3 min and analyzed by GC-MS (7890B, Agilent, Palo Alto, CA, USA), equipped with an HP-5MS column (30 m × 0.25 mm, film thickness 0.25 μm). Initial oven temperature was kept at 50 °C for 3 min, then raised to 160 °C at 3 °C/min, and further raised to 220 °C at 10 °C/min, finally kept at 220 °C for 2 min. The carrier gas was helium (He) of ultra-high purity (from a local distributor) with the flow velocity adjusted at 1 mL/min. The mass spectrometer was operated in the positive electron ionization mode at 70 eV and 230 °C with a scan from 50 to 500 *m*/*z*. The mass spectra of VOCs were matched with the NIST 14 Mass Spectrometry Library databases.

### 4.7. Statistical Analysis

The data were subjected to analyses of variance (ANOVA) using SPSS ver.19.0 statistical software (SPSS, Chicago, IL, USA). Duncan’s multiple-range test was employed to assess differences among treatments at *p* < 0.05.

## Figures and Tables

**Table 1:** Quantification of deformed structures of *P. litchii* caused by treatment of wheat seed culture of *S. fimicarius* BWL-H1, during infection process.

Condition	Inoculation on Untreated Leaves	*P. litchii* Inoculation on Leaves Treated with 12 g/L Wheat Seed Culture of *S. fimicarius* BWL-H1
IH% (6 hpi)	78.74% ± 0.79% (*n* = 66)	9.84 ± 1.33% * (*n* = 43)
DAP% (9–12 hpi)	---	77.11 ± 5.58% (*n* = 48)
DH% (24–48 hpi)	---	79.70 ± 6.56% (*n* = 54)
	---	*P. litchii* inoculation on leaves treated with 16 g/L wheat seed culture of *S. fimicarius* BWL-H1
DSM% (3–48 hpi)	---	83.33% ± 3.33% (*n* = 25)

Notes: * statistically significant (*p* < 0.01). Mean ± S.E. percentage number was calculated based on three independent biological repeats. *n* refers to total number of structures observed and quantified in each instance.

**Table 2:** Volatile compounds identified from 14 d culture of *S. fimicarius* BWL-H1.

Tentatively Identified Compounds ^a^ (CAS Number)	RT ^b^ (min)	Area (%)
*o*-Methoxyaniline (90-04-0)	18.8833	4.1906
Methyl anthranilate (134-20-3)	26.1807	0.4783
α-Copaene (3856-25-5)	27.6194	0.5516
Geosmin (19700-21-1)	28.6784	10.8232
*cis-*Caryophyllene (13877-93-5)	29.4235	5.202
(+)-Calarene (1733-45-5)	29.9298	7.3629
Germacrene D (37839-63-7)	31.9711	5.9553
γ-Cadinene (39029-41-9)	32.6238	0.9447
α-Muurolene (10208-80-7)	32.8013	4.222
*trans*-Calamenene (6617-49-8)	33.6999	3.8527
1,6-Dimethyl-4-(1-methylethyl)-(1,2,3,4,4a,7)hexahydronaphthalene (16728-99-7)	34.0353	5.709
Caryophyllenyl alcohol (472-97-9)	35.4697	2.2033

Notes: ^a^ Minor compounds were detected in autoclaved wheat seeds but not included in the table; ^b^ RT, retention time.

**Table 3:** Volatile compounds identified from 21 d culture of *S. fimicarius* BWL-H1.

Tentatively Identified Compounds ^a^ (CAS Number)	RT ^b^ (min)	Area (%)
Phenylethyl Alcohol (60-12-8)	15.9498	23.013
*o*-Methoxyaniline (90-04-0)	18.8742	4.6636
Methyl salicylate (9041-28-5)	19.6214	3.5798
2,6-Dimethoxyphenol (91-10-1)	22.0447	0.7572
2-Methoxy-6-methylaniline (50868-73-0)	22.4178	0.77
Ethyl phenylacetate (101-97-3)	22.475	3.4846
Methyl anthranilate (134-20-3)	26.1925	4.4317
α-Copaene (3856-25-5)	27.6234	6.1812
*trans*-1,10-Dimethyl-*trans*-9-decalol (19700-21-1)	28.6737	4.3395
(+)-Calarene (1733-45-5)	29.0428	1.2989
Caryophyllene (87-44-5)	29.4244	3.1322
4-Ethylphenol (123-07-9)	29.5647	3.9247
(+)-Calarene (1733-45-5)	29.9329	9.3446
Humulene (6753-98-6)	30.807	3.2429
1,5,9,9-Tetramethyl-1,4,7-cycloundecatriene (N.A. ^c^)	30.8328	1.4562
Germacrene D (37839-63-7)	31.9703	1.6972
α-Muurolene (10208-80-7)	32.7906	3.2963
*cis*-Calamenene (6617-49-8)	33.7001	2.6585
1,6-Dimethyl-4-(1-methylethyl)-(1,2,3,4,4a,7)hexahydronaphthalene (16728-99-7)	34.0368	7.2795
Caryophyllenyl alcohol (472-97-9)	35.4702	0.6922

Notes: ^a^ Minor compounds were detected in autoclaved wheat seeds but not included in the table; ^b^ RT, retention time; ^c^ N.A., CAS number Not Available.

**Table 4:** Volatile compounds used for individual test against *P. litchii* in vitro.

Compound	Source	Purity
Phenylethyl alcohol	Aldrich	≥99.0% (GC)
Methyl salicylate	Sigma–Aldrich	≥99.0% (GC)
Ethyl phenylacetate	Sigma–Aldrich	≥98.5% (GC)
Methyl anthranilate	Aldrich	≥98%, FCC, FG
α-Copaene	Sigma–Aldrich	≥99% (GC)
Caryophyllene	Sigma	≥98.5% (sum of enantiomers, GC)
4-Ethylphenol	Sigma–Aldrich	≥99% (GC)
Humulene	Aldrich	≥96.0% (GC)

**Table 5:** Effect of volatile compounds on *P. litchii* mycelial growth.

Volatile Compound	Mycelial Growth (mm) at Different Concentrations of Volatiles					
	0 µL/L	2 µL/L	20 µL/L	200 µL/L	500 µL/L	1000 µL/L
Phenylethyl alcohol	51.0a	46.1 ± 0.2b	45.7 ± 0.1b	39.1 ± 0.2c	38.7 ± 0.2c	38.1 ± 0.2c
Methyl salicylate	51.0a	47.8 ± 0.2a	21.8 ± 0.2b	0.0c	0.0c	0.0c
Ethyl phenylacetate	51.0a	47.5 ± 0.1a	11.8 ± 0.3b	9.2 ± 0.1c	0.0d	0.0d
Methyl anthranilate	51.0a	46.5 ± 0.2b	23.8 ± 0.2c	18.2 ± 0.3d	15.8 ± 0.4d	12.3 ± 0.2e
α-Copaene	51.0a	49.6 ± 0.3a	49.2 ± 0.1a	12.4 ± 0.2b	0.0c	0.0c
Caryophyllene	51.0a	49.3 ± 0.2a	43.6 ± 0.4b	22.6 ± 0.2c	20.4 ± 0.1c	18.4 ± 0.3c
4-Ethylphenol	51.0a	4.1 ± 0.2b	3.8 ± 0.1b	2.2 ± 0.1b	1.7 ± 0.2b	0.0b
Humulene	51.0a	51.2 ± 0.2a	50.8 ± 0.3a	50.1 ± 0.2a	49.7 ± 0.3a	49.5 ± 0.2a

Means based on 27 replicates followed by the same letters within the column were not significantly different (*p* < 0.05) according to the Least Significant Difference test.

**Table 6:** Effect of volatile compounds on *P. litchii* sporulation.

Volatile Compound	Sporulation (×10^4^ Spores Per Plate) at Different Concentrations of Volatiles					
	0 µL/L	2 µL/L	20 µL/L	200 µL/L	500 µL/L	1000 µL/L
Phenylethyl alcohol	132.8 ± 22a	134.2 ± 1a	133.4 ± 13a	63.6 ± 2b	61.3 ± 2b	58.5 ± 4b
Methyl salicylate	132.8 ± 22a	137.4 ± 7a	52.8 ± 3b	0.0c	0.0c	0.0c
Ethyl phenylacetate	132.8 ± 22a	127.5 ± 3b	00c	0.0c	0.0c	0.0c
Methyl anthranilate	132.8 ± 22a	136.3 ± 4a	20.1 ± 2b	18.4 ± 2b	17.5 ± 1b	7.6 ± 1c
α-Copaene	132.8 ± 22a	134.6 ± 9a	132.3 ± 6a	17.7 ± 2b	0.0c	0.0c
Caryophyllene	132.8 ± 22a	138.3 ± 5a	95.4 ± 12b	6.1 ± 13c	2.9 ± 4c	0.0c
4-Ethylphenol	132.8 ± 22a	0.0b	0.0b	0.0b	0.0b	0.0b
Humulene	132.8 ± 22a	133.8 ± 14a	134.6 ± 17a	132.5 ± 20a	131.8 ± 23a	131.4 ± 21a

Means based on 27 replicates followed by the same letters within the column were not significantly different (*p* < 0.05) according to the Least Significant Difference test.

**Table 7:** Effect of volatile compounds on *P. litchii* sporangia germination.

Volatile Compound	Sporangia Germination (%) at Different Concentrations of Volatiles					
	0 µL/L	2 µL/L	20 µL/L	200 µL/L	500 µL/L	1000 µL/L
Phenylethyl alcohol	94.7 ± 0.9a	93.4 ± 0.5a	92.2 ± 0.3ab	88.9 ± 0.7b	63.8 ± 0.4c	39.8 ± 0.3d
Methyl salicylate	95.4 ± 1.1a	92.6 ± 0.8b	89.7 ± 1.3c	0.0c	00.0c	0.0c
Ethyl phenylacetate	94.9 ± 0.7a	94 ± 1.0a	90.8 ± 0.9ab	87.7 ± 1.2b	68.4 ± 0.3c	41.3 ± 0.2d
Methyl anthranilate	95.1 ± 1.1a	92.8 ± 1.4a	91.5 ± 0.8ab	88.2 ± 0.7b	38.8 ± 0.2c	0.0d
α-Copaene	94.7 ± 0.9a	91.3 ± 0.7a	78.6 ± 0.9b	0.0c	0.0c	0.0c
Caryophyllene	95.4 ± 1.1a	92.7 ± 1.2ab	92.4 ± 0.8ab	88.2 ± 0.3b	52.6 ± 0.2c	27.6 ± 0.2c
4-Ethylphenol	95.1a ± 1.1a	23.9b ± 0.1b	0.0c	0.0c	0.0c	0.0c
Humulene	94.9a ± 0.7a	93.7 ± 1.5ab	92.6 ± 2.0ab	91.5 ± 1.8ab	91.4 ± 1.5ab	91.2 ± 1.2b

Means based on 27 replicates followed by the same letters within the column were not significantly different (*p* < 0.05) according to the Least Significant Difference test.

**Table 8:** Effect of volatile compounds on *P. litchii* germ tube growth.

Volatile Compound	The Length of Germ Tube (µm) at Different Concentrations of Volatiles					
	0 µL/L	2 µL/L	20 µL/L	200 µL/L	500 µL/L	1000 µL/L
Phenylethyl alcohol	251.3 ± 35.4a	151.0 ± 2.5b	75.6 ± 3.1c	68.4 ± 4.1c	19.2 ± 2.5d	18.2 ± 1.9d
Methyl salicylate	244.6 ± 8.8a	142.7 ± 2.0b	123.9 ± 3.4c	0.0d	0.0d	0.0d
Ethyl phenylacetate	254.6 ± 44.0a	202.8 ± 4.7b	198.3 ± 2.7b	87.7 ± 2.3c	73.6 ± 2.0c	65.2 ± 5.6c
Methyl anthranilate	241.3 ± 8.8a	137.7 ± 3.4b	119.4 ± 2.4c	62.8 ± 3.5d	23.4 ± 3.6e	0.0f
α-Copaene	251.3 ± 35.4a	49.7 ± 2.4b	26.8 ± 2.3bc	0.0c	0.0c	0.0c
Caryophyllene	244.6 ± 8.8a	195.1 ± 5.0b	151.4 ± 4.2c	99.3 ± 4.5d	30.2 ± 3.8e	18.4 ± 3.9f
4-Ethylphenol	241.3 ± 8.8a	36 ± 3.1b	0.0c	0.0c	0.0c	0.0c
Humulene	254.6 ± 44.0a	256.9 ± 5.7a	234.3 ± 3.3ab	230.5 ± 0.7ab	229.4 ± 2.1ab	220.5 ± 2.7b

Means based on 27 replicates followed by the same letters within the column were not significantly different (*p* < 0.05) according to the Least Significant Difference test.

## Supplementary Materials

The following are available online.
