# Supplementary material for: Antifungal Activity of Natural Volatile Organic Compounds against Litchi Downy Blight Pathogen Peronophythora litchii
Source: Molecules. 2018 Feb 8;23(2):358. doi: 10.3390/molecules23020358 (PMC6017977; doi:10.3390/molecules23020358)
Supplement: Supplementary file 1 [file molecules-23-00358-s001.pdf]

Supplementary material

Antifungal activity of natural volatile organic compounds against litchi downy blight pathogen *Peronophythora litchii*

Mengyu Xing, Li Zheng, Yizhen Deng, Dandan Xu, Pinggen Xi, Minhui Li, Guanghui Kong and Zide Jiang*

Department of Plant Pathology/Guangdong Province Key Laboratory of Microbial Signals and Disease Control, South China Agricultural University, Guangzhou 510642, China

***Corresponding author** Zide Jiang

Complete postal address Department of Plant Pathology/Guangdong Province Key Laboratory of Microbial Signals and Disease Control, South China Agricultural University, Wushan Road, No. 438, Tianhe District, Guangzhou 510642, China.

Phone number +86-020-38604779

E-mail address zdjiang@scau.edu.cn

Fax number +86-020-85280203

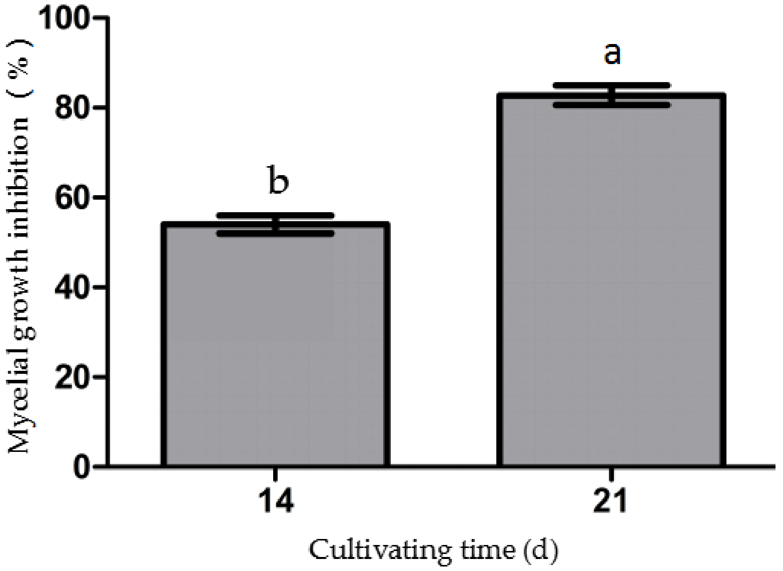

Figure s1. Suppression of *P. litchii* mycelial growth by volatiles from 14 d or 21 d culture of *S. fimicarius* BWL-H1. *P. litchii* was fumigated with 16 g/L of 14 d or 21 d wheat seeds culture with *S. fimicarius* BWL-H1. Mycelial growth inhibition was calculated according to the mycelial diameter cultured after 6 days at 25 °C. Mycelial growth inhibition fumigated with 21 d cultures and 14 d cultures was 82.8% and

54.1% respectively. Data are mean ± SE of 27 replicates of three repetitions of the experiment. Values followed by different letters were significantly different ($P<0.05$).
